# Transient Receptor Potential Cation Channels in Cancer Therapy

**DOI:** 10.3390/medsci7120108

**Published:** 2019-11-30

**Authors:** Giorgio Santoni, Federica Maggi, Maria Beatrice Morelli, Matteo Santoni, Oliviero Marinelli

**Affiliations:** 1 School of Pharmacy, University of Camerino, via Madonna delle Carceri 9, 62032 Camerino (MC), Italy; federica.maggi@uniroma1.it (F.M.); mariabeatrice.morelli@unicam.it (M.B.M.); oliviero.marinelli@unicam.it (O.M.); 2 Department of Molecular Medicine, Sapienza University, Viale Regina Elena 324, 00161 Rome (RM), Italy; 3 Medical Oncology Unit, Hospital of Macerata, Via Santa Lucia 2, 62100 Macerata (MC), Italy; mattymo@alice.it; 4 School of Biosciences and Veterinary Medicine, University of Camerino, via Madonna delle Carceri 9, 62032 Camerino (MC), Italy

**Keywords:** transient receptor potential channels, tumor progression, chemotherapy resistance

## Abstract

In mammals, the transient receptor potential (TRP) channels family consists of six different families, namely TRPC (canonical), TRPV (vanilloid), TRPM (melastatin), TRPML (mucolipin), TRPP (polycystin), and TRPA (ankyrin), that are strictly connected with cancer cell proliferation, differentiation, cell death, angiogenesis, migration, and invasion. Changes in TRP channels’ expression and function have been found to regulate cell proliferation and resistance or sensitivity of cancer cells to apoptotic-induced cell death, resulting in cancer-promoting effects or resistance to chemotherapy treatments. This review summarizes the data reported so far on the effect of targeting TRP channels in different types of cancer by using multiple TRP-specific agonists, antagonists alone, or in combination with classic chemotherapeutic agents, microRNA specifically targeting the TRP channels, and so forth, and the in vitro and in vivo feasibility evaluated in experimental models and in cancer patients. Considerable efforts have been made to fight cancer cells, and therapies targeting TRP channels seem to be the most promising strategy. However, more in-depth investigations are required to completely understand the role of TRP channels in cancer in order to design new, more specific, and valuable pharmacological tools.

## 1. Introduction

Despite advances in findings and medical care for various cancers, there are still high rates of treatment failure and mortality worldwide. This high frequency is mainly due to the powerful capability of cancer cells to proliferate and migrate. Recent studies regarding transient receptor potential (TRP) channels have indicated that they mark a specific phase of cancer progression and that they could represent potential targets for cancer treatment. TRP channels are important as calcium- permeable and non-selective ion channels expressed in different tissues and cell types in mammals and are crucial regulators of calcium, sodium, and magnesium ions. They are grouped into six subfamilies: TRPC (“C” for canonical), TRPV (“V” for vanilloid), TRPM (“M” for melastatin), TRPA (“A” for ankyrin), TRPP (“P” for polycystic), and TRPML (“ML” for mucolipin) [1]. They show common features in structure, such as the presence of six transmembrane segments with intracellular N- and C-terms and various degrees of sequence homology. TRP subfamilies have been linked to many physiological and pathological functions, including cell differentiation, proliferation, apoptosis, and ionic homeostasis.

Upregulation and downregulation in TRP channel subfamily expression are linked with the different phases of tumor progression and strongly correlate with clinical parameters (e.g., overall survival (OS), progression-free survival, and more) as shown by Kaplan–Meier, univariate, and multivariate Cox regression analysis. Indeed, high TRPM8 expression is related to a worse OS in pancreatic cancer (PC) patients (*p* = 0.001) [2]; and increased TRPM7 expression represents an unfavorable factor in human bladder cancer (BCa) (*p* < 0.05) [3]. In esophageal squamous cell carcinoma (OSCC), TRPM7 expression represents an independent prognostic factor of good post-operative survival (*p* < 0.05) [4], whereas TRPV6 downregulation is associated with an unfavorable 3-year disease-specific survival (*p*  =  0.027) [5]. Overexpression of TRPV3 correlates with tumor progression and short OS in non-small cell lung carcinoma (NSCLC) (*p* = 0.020) [6]; and loss or reduction of TRPML1 mRNA expression correlates with short survival in glioblastoma (GBM) patients (*p* < 0.0298) [7]. In addition, in diffuse large B cell lymphoma, TRPM4 positivity confers worse OS (*p* = 0.004) and progression-free survival (*p* = 0.005) in rituximab-, cyclophosphamide-, doxorubicin-, vincristine-, and prednisone-treated lymphoma cells [8].

Therefore, TRP channels represent promising potential diagnostic, prognostic, and therapeutic tools for different types of cancer [9,10]. In this review, we report the results regarding the in vitro and in vivo therapeutic approach with different compounds that affect the expression and functions of TRP channels in cancer therapy.

## 2. TRPC Channels in Cancer Therapy

Several channels belonging to the TRPC subfamily have been found to be a target in cancer therapy. Treatment of colorectal cancer (CRC) cells with 10 μM of 20-*O*-β-d-glucopyranosil-20(*S*)-protopanaxadiol (20-GPPD), a metabolite of ginseng saponin, induces activation of Ca^2+^ influx through TRPC channels, leading to apoptosis [11]. Treatment of CT-26 murine colon cancer cells with 20-GPPD for 24 h triggers an accumulation of cells in the sub-G1 cell phase and apoptosis. In addition, this compound is able to stimulate the phosphorylation of AMP-activated protein kinase (AMPK), leading to AMPK activation and consequently inhibition of cell viability. The 20-GPPD induces an increase in AMPK activation, triggered by TRPC-dependent enhancement in intracellular Ca^2+^ concentration ([Ca^2+^]_i_), as demonstrated by the capability of the TRPC non-selective blockers, Gd^3+^ and SKF96365, to completely inhibit Ca^2+^ influx.

Several GBM cell lines express different members of the TRPC channels (e.g., TRPC1, 3, 4 and 5). Sustained treatment of glioma cells with the inhibitors SKF96365 (25 μM) and aminoethoxydiphenyl borate (2-APB) (100 μM) reduced cell proliferation, with an accumulation of glioma cells in the G2/M phase and impairment of cytokinesis [12,13]. However, 2-APB is not only a TRPC inhibitor, but it can also activate both TRPV2 and TRPV3 and, in addition, works as an inositol triphosphate (IP_3_)-induced Ca^2+^-release inhibitor. However, data on cell viability should be confirmed in TRPC1-silenced cells to verify the specific role of this channel. On the other hand, a role for TRPC1 in glioma cell chemotaxis induced by epidermal growth factor (EGF) has been demonstrated [14]. In this regard, in GBM cells, TRPC1 silencing or blockade using SKF96365 at 25 μM, 2-APB at 100 μM, or MRS1845 at 25 μM inhibits the chemotactic migration induced by EGF but not basal migration. The localization of TRPC1 in lipid rafts has been found to be essential for TRPC function as well as for EGF-induced chemotaxis [13].

The inhibition of TRPC1 using 2-APB or TRPC1 silencing reduces the adhesive and invasive capabilities of nasopharyngeal cancer cells, suggesting that TRPC1 can modulate metastasis spreading [15].

The sesquiterpene (-) Englerin A (EA, 250 nM) from the plant *Phyllanthus engleri* induces cytotoxicity in different cancer types, including renal cell carcinoma (RCC), but not in normal cells. A common feature in RCC lines is the expression of heteromeric TRPC1/C4/C5 channels. TRPC4 expression is required for EA-induced calcium influx, membrane depolarization, and growth inhibition. EA is a TRPC4 agonist; however it also activates TRPC1/C5 channels. TRPC4 stimulation in cancer cells induced growth inhibition, which can be blocked by ML204, a TRPC4/C5 inhibitor. EA also weakly inhibits the TRPA1, TRPV3/V4, and TRPM8 channels, suggesting that it can bind a common domain present in the TRP ion channels [16].

Triple-negative breast cancers (TNBCs) are an aggressive heterogeneous group of tumors resistant to several target therapies, resulting in high relapse and poorer OS. A recent report has identified a group of TNBC cell lines responsive to EA treatment. The BT-549 and Hs578T TNBC BC cell lines, which express high TRPC4 and TRPC1/C4 heterodimer levels, are more sensitive to EA than other TNBC cell lines. In Hs578T TNBC cells, EA induces Na^+^ and Ca^2+^ accumulation, whereas in BT-549 cells, it increases cytosolic Ca^2+^ levels and induces mitochondrial depolarization [17]. In human SW982 synovial sarcoma cells (SSCs), EA induces TRPC1/C4 heterodimer activation and cell cytotoxicity, which is inhibited by Pico145, an inhibitor of the TRPC1/C4 channels. EA cytotoxicity is due to TRPC1 or TRPC4 suppression. Ouabain (10 nM), an Na^+^/K^+^-ATPase inhibitor, increases EA-induced cytotoxicity; Na^+^ entry by the Na^+^ loading ionophore, gramicidin-A, causes cell death of SW982 cells, which are resistant to Pico145 (10 nM), suggesting that Na^+^ loading is itself cytotoxic even without TRPC1/C4 activation. Overall, these results evidenced that EA-mediated cytotoxicity in human SSCs depends both on TRPC1/C4 channels and Na^+^ loading [18].

EA exerts a rapid cytotoxic effect on TRPC4-positive A498 RCCs and Hs578T TNBC. Different members of the TRP channel family have been found to assembly to form homo- and heterodimers [16,17]. Regarding the effect of EA, it is mediated by TRPC1/TRPC4 heterodimers and both TRPC4 and TRPC1 are required; however, although TRPC4 was necessary for the EA-evoked Ca^2+^ elevation, TRPC1 negatively regulated Ca^2+^ entry. By contrast, both TRPC4 and TRPC1 were necessary for monovalent cation entry evoked by EA, and EA-evoked cell death was dependent upon entry of Na^+^. Therefore, it can be hypothesized that Na^+^/K^+^-ATPase might protect cells by counteracting the sustained Na^+^ entry. Indeed, inhibition of Na^+^/K^+^-ATPase by ouabain increases the EA-evoked cytotoxicity, suggesting that EA-mediated cancer cell cytotoxicity sustains Na^+^ entry through the heteromeric TRPC1/TRPC4 channels and EA cytotoxicity can be increased by Na^+^/K^+^-ATPase inhibition [19].

The diterpene ester tonantzitlolone (TZL) is a natural product, which shows at a nanomolar dose cytotoxicity toward RCCs. Although chemically distinct to EA, its effects are similar to other drugs that target TRPC1/4/5 channels. TZL enhances the intracellular Ca^2+^ and induces TRPC4 and TRPC5 overexpression and the assembly of TRPC1-TRPC4 and TRPC1-TRPC5 heterodimers in A498 RCC line, which are inhibited by Pico145. No activated endogenous store-operated Ca^2+^ entry (SOCE) or TRPC3, TRPV4, or TRPM2 overexpression is induced by TZL in HEK293 cells [20].

An analogue of the xanthine-based Pico145 inhibitor, AM237 at 15 to 20 nM, activates TRPC5 in the A498 RCC line and potentiates TRPC5 activation by sphingosine-1-phosphate. AM237 also activates TRPC5 channels and potently inhibits EA-dependent activation [21]. By contrast, it did not activate TRPC4-C4, TRPC1-C4, and TRPC1-C5 channels.

MTI-101 is a peptidomimetic compound that binds the CD44/Integrin Subunit Alpha 4 complex and triggers necrosis in multiple myeloma (MM) cell lines. The MTI-10 stimulates an increase in intracellular Ca^2+^ levels in MM cells. Pharmacological inhibition of store-operated channels or reduction in TRPC1 expression blocks MTI-101-induced death. The effect of MTI-101 in MM is more evident in relapsed myeloma patients, suggesting an increased sensitivity to Ca^2+^ overload-mediated cell death in relapsed MM. Moreover, MTI-101 synergized with bortezomib in both MM cell lines and primary myeloma patients [22].

In NSCLC, the expression of TRPC1, C3, C4, and C6 channels correlates with tumor differentiation grade [23]. Treatment of A549 cells with GBM cells with SKF96365 (25 μM) and 2-APB (100 μM), all-trans retinoic acid (ATRA, 1 μM) for 96 h increased TRPC3, C4, and C6 expression and enhanced Ca^2+^ influx. The A549 cell proliferation is more sensitive to 2-APB, following chronic application of ATRA [23]. However, ATRA shows no direct effect on TRPC channel function, and the increased Ca^2+^ influx is probably due to *TRPC* gene upregulation.

The SOCE controls the proliferation and metastasis of several cancer types. Their role in nicotine-promoted proliferation of NSCLC A549 cells has been investigated. A549 cells express high levels of TRPC1 and C6 and Orai1 store-operated calcium channels and low TRPC3 and TRPC4 levels. Nicotine at 1 μM upregulates TRPC1, TRPC6, Orai1, mRNA, and protein expression; increases basal [Ca^2+^]_i_; and enhances SOCE. The promotion of cell proliferation is observed in nicotine-treated cells, which is inhibited by the SOCE inhibitor, SKF-96365. Furthermore, nicotine upregulates hypoxia inducible factor 1α (HIF-1α) expression in A549 and NCI-H292 cells; silencing of HIF-1α stimulates an increase in TRPCs and Orai1 and decreases basal [Ca^2+^]_i_ and SOCE. In conclusion, nicotine promotes lung cancer cell proliferation by upregulating HIF-1α and Orai1 store-operated calcium channel components and therefore enhancing SOCE and increasing [Ca^2+^]_I_ [24].

Abnormal expression of TRPC5 has been associated with cancer progression and chemoresistance. A role for the overexpression of TRPC5 and P-glycoprotein (P-gp) in adriamycin-resistant (ADMR) MCF-7 BC cells has been demonstrated [25]. The TRPC5 expression is higher in ADMR-MCF-7 cells compared to controls. A marked downregulation of P-gp expression and an increase of adriamycin accumulation was evidenced in ADMR-MCF-7 cells using an anti-TRPC5 T5E3 antibody. In addition, miR-230a, which specifically targets TRPC5 and nuclear factor of activated T-cells 3 (NFATc3) mRNAs, is found to be downregulated in ADMR-MCF-7 cells, suggesting its involvement in chemoresistance, and a low expression of miR-230a is associated with poor patient outcomes. Finally, in regard to NFATc3, but not the other NFAT isoforms, it stimulates the transcriptional activity of the promoter of Multi Drug Reactivity 1 gene, suggesting that Ca^2+^entry through TRPC5 stimulates NFATc3 to enhance P-gp production [25].

Cancer cells produce soluble factors and secrete extracellular vesicles (EVs), including exosomes, microvesicles, and apoptotic bodies [26]. TRPC5 is involved in EVs’ formation and secretion in ADMR-MCF-7 [27]. TRPC5 is packaged in EVs and transported in recipient cells, where it promotes P-gp expression by increasing calcium (Ca^2+^) influx. EVs levels containing TRPC5 are related to the acquired chemoresistance [26]. Zhang and colleagues also demonstrated that TRPC5 regulates chemotherapy-induced autophagy in BC cells via calcium/calmodulin-dependent protein kinase, AMPKα, and the mammalian target of rapamycin pathway. TRPC5-induced autophagy functions as a pro-survival mechanism promoting chemoresistance [28]. Overexpression of TRPC5 is also involved in 5-Fluorouracil (5-Fu) resistance in CRC [29]. Previously it has been reported that TRPC5 mediated Ca^2+^entry, stimulating the ATP-binding cassette, subfamily B, member 1 (ABCB1) pump overproduction in drug-resistant BC cells, and suppressing TRPC5, reversing drug resistance through the TRPC5-NFATc3-Ca^2+^-ABCB1 pathways [25]. TRPC5 and the ABCB1 pump are also found to be upregulated in human-resistant HCT-8 and LoVo CRC cell lines. TRPC5 activation promotes β-catenin translocation in the nucleus, increases glycolysis and ATP production, and stimulates the expression of ABCB1 and cyclin D1 proteins, contributing to 5-Fu resistance. Thus, suppressing TRPC5 expression weakens the ABCB1 pump and causes a reversal of 5-Fu resistance in HCT-8/5-Fu and LoVo/5-Fu cells. High expression of TRPC5 is associated with upregulation of glucose transporter 1 in CRCs, and an increase in glycolysis occurs in chemoresistance cells [30].

TRPC6 channels are essential for the G2/M phase transition in gastric cancer (GaC) and OSCC [31,32]. In these studies, SKF96365 (10 μM) blocks TRPC6 channel activity, leading to cell growth arrest and accumulation in the G2/M phase. The specific role of TRPC6 channels is confirmed by the heterologous expression of a dominant negative of TRPC6 in GaC cells. The regulation of cancer cell proliferation by TRPC6 involves an elevation of [Ca^2+^]i that is essential for G2/M phase transition in GaCs and OSCCs.

In hepatocellular carcinoma (HCC), doxorubicin chemoresistance occurs through epithelial mesenchymal transition (EMT) induction, with vimentin upregulation and downregulation of E-cadherin and claudin1 EMT-associated proteins. Long treatment with doxorubicin, increasing Ca^2+^ influx, stimulates chemoresistance, which promotes EMT. The TRPC6 channel induces EMT by signal transducer and activator of transcription 3 activation, which is a downstream regulator in the TRPC6 calcium signaling pathway. In an HCC xenograft model, TRPC6 silencing resulted in slower growth and greater doxorubicin sensitivity with respect controls [33].

Data relative to TRPC channel-related effects are synthetized in Table 1.

## 3. TRPM Channels in Cancer Therapy

Among the TRPM family, an important antitumor effect has been mediated by TRPM2, TRPM5, TRPM7, and TRPM8 channels.

The histone deacetylase inhibitors Tricostatin A (100 nM) and sodium butyrate (5 mM) induce TRPM2 upregulation and apoptosis in a TRPM2-dependent manner in T24 BCa cells. The TRPM2 upregulation induced by histone deacetylases inhibition is due to an enhancement of acetylated H3K9 in the TRPM2 promoter. A previous report on TRPM2 modulators evaluated in *Xenopus* oocytes evidenced that H_2_O_2_, AMP, cyclic ADPR, dinucleotide phosphate, and nicotinic acid adenine dinucleotide do not affect TRPM2 channels in physiological conditions [34]. 5-Fu and Leucovorin (LCV) are widely used in BC and CRC for chemotherapy. It has been reported that the expression of TRPM2 channels increases in cancer cells. In BC (MCF-7) and CRC (Caco-2) cell lines, 5-Fu (5 μM) and LCV (2 μM), in combination or alone, result in TRPM2 activation with increased [Ca^2+^]i levels and oxidative stress-induced apoptosis. The effects of 5-Fu and LCV is directly related to TRPM2. These channels play an important role in the apoptosis of cancer cells by elevating Ca^2+^ and intracellular reactive oxygen species (ROS) levels and mitochondrial depolarization [35].

A recent report evidenced that Selenium (Se, 1 μM) enhances the apoptotic efficacy of docetaxel (DTX, 10 nM), through the activation of TRPM2 in the DBTRG GBM cell line [36]. DTX induces cancer cell death through excessive ROS production and increases Ca^2+^ entry. TRPM2 activated by ROS and Se stimulates the apoptosis of DTX-resistant GBM cells. DTX and Se in combination induce mitochondrial membrane depolarization and ROS production, and increase NAD-dependent DNA repair enzyme poly (ADP-ribose) polymerase-1 (PARP-1) activity and apoptosis in DBTRG cells.

A recent report of Maeda and collaborators discusses a role of TRPM5 in lung metastasis [37]. Acid extracellular pH has been found to increase intracellular Ca^2+^and matrix metalloproteinase-9 (MMP-9) expression in the mouse B16 melanoma (ME) model. TRPM5 silencing reduces extracellular acid-induced MMP-9 expression, whereas enforced TRPM5 expression shows the opposite effect as well as increasing lung metastasis. Treatment of ME-bearing mice with the TRPM5 inhibitor triphenylphoshine oxide reduces spontaneous lung metastasis. Moreover, in ME and GaC patients, high TRPM5 mRNA expression correlates with poor OS rates.

TRPM7 has been found to regulate BC cell proliferation, migration, invasion, and metastasis; however, the effect of the TRPM7 kinase domain in the control of BC migration and invasion has only recently been evaluated by using a TRPM7 kinase assay and a new TRPM7 kinase inhibitor TG100-115. This inhibitor shows little effect on the proliferation of MDA-MB-231 on BCs, but significantly inhibits the migration and invasion of BC cells as a consequence of reduced myosin IIA heavy chain and focal adhesion kinase (FAK) phosphorylation. TG100-115 also suppressed the TRPM7 channel activity [38].

Waixenicin A (WA) is an extract from *Sarcotheliaedmondsoni* (syn. *Anthelia edmondsoni*), a soft coral from Hawaii, that inhibits TRPM7 in a dose-dependent manner [39]. Moreover, WA (10 μM) completely inhibits the TRPM7 current in TRPM7-transfected HEK293 cells. The inhibitory effects of WA on TRPM7 are strongly dependent on [Mg^2+^]_i_, indicating that this compound enhances Mg^2+^ blockade of the channels or that Mg^2+^ enhances the binding affinity of WA. Importantly, WA does not exert an effect on TRPM6 channels, the closest homologues of TRPM7 channels. Zierler et al. showed that WA at 50 μM inhibits the proliferation of human Jurkat T cells and rat basophilic leukemia cells in a TRPM7-dependent manner [39]. Recently, Kim et al. demonstrated that WA is a potent inhibitor of GaC and BC proliferation in a TRPM7-dependent manner [40,41]. Indeed, WA is able to inhibit AGS GaC cell and MCF-7 BC cell proliferation as well as reduce the TRPM7 currents in these cell lines. Moreover, ginsenoside Rd, one of the more active ginseng saponin components, used at 500 μM, has been shown to block TRPM7 channels and to induce cell death in GaC and BC cells [41]. Indeed, ginsenoside Rd decreases cell viability in MCF-7 and AGS in a dose-dependent manner, while it increases cell viability in HEK293 cells. Ginsenoside Rd-induced cell death is due to intrinsic apoptosis signaling via mitochondrial membrane depolarization. Moreover, ginsenoside Rd increases caspase-3 activity in both MCF-7 and AGS cancer cells. Importantly, ginsenoside Rd inhibits TRPM7 currents in MCF-7 and AGS cells.

Recent findings in glioma have shown that vacquinol-1 (Vac) promotes cell death as a consequence of inefficient vacuole–lysosome fusion, which is reversed by exogenous ATP in GBM [42]. Exogenous ATP activates TRPM7, Ca^2+^and Mg^2+^ influx and phosphoinositide 3-kinases (PI3K) activation that promotes vesicle fusion with lysosomes. Thus, the inhibitory effect on ATP-mediated cell death induced by Vac depends to TRPM7 activation that stimulates the PI3K pathway, restoring vacuole–lysosome fusion. Overexpression of TRPM7 is responsible for Vac resistance in glioma cells. TRPM7 is required to prevent apoptosis in PC. Silencing TRPM7 in PC cells induces the replicative senescence program [43]. Downregulation of TRPM7 enhances gemcitabine cytotoxicity in PC [43].

TRPM8 is expressed or overexpressed in different cancer types (e.g., CRC, PCa, OSCC, and BC). Tsaveler et al. originally identified *TRPM8* by screening a prostate cDNA library; the gene was described as a novel prostate-specific gene with increased expression during the transformation of PCa [44]. Regarding agonists or antagonists of TRPM8 channels, several are now considered in cancer prevention and therapy, although some of those reported in the literature lack selectivity for TRPM8 because they also act on TRPV1 and TRPA1 [45,46].

In normal prostate cells, there is a slight level of TRPM8 expression, while in PCa, the expression of TRPM8 is increased [47]. Asuthkar et al. demonstrated that TRPM8 is an ionotropic testosterone receptor [48]. In early PCa tumors with high androgen levels, TRPM8 is expressed, while anti-androgen therapy reduced its expression [49]. Although TRPM8 mRNA is expressed at high levels, TRPM8 protein undergoes ubiquitination and degradation in PCa cells. Overexpression of TRPM8 induces anti-proliferation and pro-apoptotic effects. The cell cycle arrest and reduced cell motility is through downregulation of Cdk4/6 and focal adhesion kinase (FAK), respectively [50]. Treatment of PCa cells with TRPM8 agonist menthol accompanied by androgen receptor (AR) inhibition or TRPM8 overexpression, respectively, showed greater anti-proliferative effect [48]. Furthermore, the TRPM8 agonist WS12 encapsulated into lipid nanocapsules is able to impair cancer cell migration ability [51]. On the other hand, testosterone is able to inhibit TRPM8 activity [52]. Indeed, low (10 nM), but not high (100 nM), testosterone concentrations decrease TRPM8-mediated Ca^2+^ influx, resulting in a significant increase in cell migration. This process is induced by TRPM8/AR colocalization in lipid raft microdomains of the plasma membrane, where AR inhibits TRPM8 activity. As a result, increased FAK phosphorylation leads to PCa cell migration.

In the T24 BCa cell line, menthol (1 mM) induces cell death [53]. At 0.1 mM augments, the migration and invasion abilities of both TRPM8-overexpressing HSC3 and HSC4 oral squamous carcinoma (OSC) cell lines were shown by potentiating the MMP-9 activity, and this effect is completely suppressed by a novel TRPM8 antagonist RQ-00203078 used at a 10-μM dose [54].

Moreover, cannabigerol (CBG), a non-psychotropic cannabis-derived cannabinoid, used at 10 μM, potently blocks TRPM8 in CRC and protects against cancer development and progression [55]. CBG promotes ROS-dependent apoptosis, upregulates CCAAT-enhancer-binding protein homologous protein (CHOP) mRNA expression, and inhibits cell growth in CRC cells. TRPM8 silencing reduces the effect of CBG on cell growth and on CHOP mRNA expression. In vivo, CBG inhibits in a TRPM8-dependent manner the growth of xenograft tumors as well as chemically induced colon carcinogenesis. Although, in CRC, CBG is able to induce TRPA1, TRPV1, and TRPV2 channel activation, its proapoptotic effects are TRPA1, TRPV1, and TRPV2 independent [55].

TRPM8 blockers, such as BCTC, clotrimazole, and DD01050, as well as more specific blockers like AMTB and JNJ41876666, have been used in different human PCa cell lines [56,57]. BCTC (10 μM), clotrimazole (10 μM), AMTB (10 μM), and JNJ41876666 (10 μM) inhibit cell proliferation in all PCa cell lines but not in normal prostate cells. Moreover, RQ-00203078, another TRPM8 antagonist, is used in HSC3 and HSC4 OSC cell lines [54]. RQ-00203078 (10 μM) completely abolishes menthol-induced TRPM8 whole-cell currents and SOCE in both cell lines. Moreover, RQ-00203078 inhibits both menthol-induced basal cell proliferation as well as menthol-induced basal migration and invasion. Menthol-induced MMP-9 activity is also suppressed by RQ-00203078 [54]. In addition, recently, tetrahydroisoquinoline-derived urea and 2,5-Diketopiperazine derivatives as selective antagonists of TRPM8 with high anti-PCa activity (at 10 nM) were synthetized by the De Petrocellis and colleagues [58].

TRPM8 is involved in cancer proliferation, invasion, and migration of LLC-2 lung cancer cells [59]. TRPM8, activating the uncoupling protein 2, induces resistance both against activated CD8^+^T lymphocytes of the spleen and doxorubicin. In PCa cells, TRPM8 enhances HIF-1α, a subunit of the transcription factor HIF-1, which promotes hypoxic growth capacity, angiogenesis, and drug resistance in cancer cells. TRPM8 promotes in vitro hypoxic growth, drug resistance, in vivo tumorigenicity, and increased HIF-1α protein levels. TRPM8-induced suppression of HIF-1α ubiquitination and enhanced HIF-1α transactivation are attenuated by forced expression of receptor of activated protein C kinase 1 (RACK1); TRPM8 overexpression reduces phospho-RACK1 levels, thus affecting its dimerization status, and promotes RACK1 binding to HIF-1α and calcineurin [60].

TRPM8 is necessary for proliferation and invasion of PCa cells and is closely related to PCa gemcitabine sensitivity [61]. In PCa cell lines, PACN-1 and BxPC-3 sensitivity to gemcitabine is increased, while proliferation and invasion have been suppressed after TRPM8 RNA interference-mediated silencing. The mechanism of TRPM8 in gemcitabine-based chemotherapy is a consequence of the reduction in the expression and activity of multidrug resistance-associated proteins, such as P-gp in response to TRPM8 silencing. Moreover, TRPM8 knockdown significantly increased human equilibrative nucleoside transporter 1 protein levels and the Bax/Bcl-2 pro-apoptotic ratio, while reducing ribonucleotide reductase M1 protein levels.

Furthermore, in osteosarcoma cells, TRPM8 knockdown induces Ca^2+^imbalance, inhibition of protein kinase B (Akt)-Glycogen synthase kinase (GSK)-3β, extracellular signal–regulated kinases (ERK)1/2 and FAK pathways, decreases proliferation, invasion, and migration, and improves apoptosis induced by epirubicin [62].

Immunotherapies might represent promising alternatives for the treatment of patients with hormone-refractory PCa. Results of phase I clinical trial reported the efficacy of vaccination with dendritic cells (DC) loaded with a cocktail consisting of HLAA*0201-restricted peptides derived from five different PCa-associated antigens prostate-specific antigen (PSA), prostate-specific membrane antigen, survivin, prostein, and also an HLA-A*0201-restricted T cell epitope derived from the PCa-associated protein TRPM8 (TRPM8 187–195, GLMKYIGEV) [63]. Eight hormone-refractory PCa patients received a total of four vaccinations every week. One patient displayed a partial response (PSA decrease >50%) and three other patients showed stable PSA values or decelerated PSA increases. Three of four PSA responders also showed antigen-specific CD8^+^T-cell activation against prostein, survivin, and prostate-specific membrane antigen. A TRPM8-restricted peptide specifically upregulated in PCa has been identified [44,49] and its ability to induce in vitro a specific T cell response and in vivo a partial response when loaded on myeloid DC cells has been demonstrated [63]. However, no increase of TRPM8-reactive CD8^+^T-lymphocytes was detected after vaccination. These results, although partially positive for TRPM8 protein, evidenced that the application of cocktail-loaded DCs induces a transient protective clinical response [64]. Further study of the molecular structure of the TRPM8 channels is required to further improve this therapeutic approach.

Microribonucleic acids (miRNAs), small non-coding RNAs of approximately 22 bp, induce RNA interference by base-pairing with the 3′ untranslated region of mRNA, which triggers either mRNA translational repression or RNA degradation [65]. Thus, miRNAs function as sequence-specific inhibitors of gene expression. miRNAs are initially transcribed as precursor transcripts called primary miRNAs. Over 1000 different miRNAs are encoded by the human genome; approximately 20% to 30% of all genes are targeted by miRNAs, and a single miRNA may target up to 200 genes [66]. In human cancers, specific miRNAs are expressed in different tissues, and changes in the control of gene expression have been associated with carcinogenesis [67]. Furthermore, miRNAs regulate the expression of different TRP genes involved in human diseases. The analysis of a possible correlation between the expression of selected miRNAs and the *TRPM8* gene have shown an inverse correlation between high TRPM8 expression and low miR-26a expression. It was found that miR-26a expression was decreased in PCa tissues and cell lines, with androgen-independent PCa showing lower miR-26a expression compared to androgen-dependent PCa [68]. Overexpression of miR-26a enhances apoptosis, and this upregulation is triggered by cytochrome c oxidase subunit II inhibition. In addition, a low miR-26a density results in an evidently poor prognosis.

TRPM3 plays a major role in the development and progression of clear cell renal cell carcinoma (ccRCC) with von Hippel-Lindau (VHL) loss mutation. TRPM3 expression is enhanced in human ccRCC with inactivated or deleted VHL. Loss of VHL inhibits the expression of miR204, that in turn leads to an increase of the oncogenic autophagy in ccRCC, resulting in augmented TRPM3 expression, a direct target of miR204 [69].

About 50% of the malignant ME show a somatic missense mutation at the amino acid residue V600 of the proto-oncogene B-raf (BRAF^600^). The BRAF inhibitor vemurafenib induces a regression of metastatic ME harboring BRAF^600^ and extracellular vesicles from vemurafenib-treated ME show increased miR211-5p expression. In BRAF^600^ ME cells, the increase of microphthalmia-associated transcription factor (MITF) that upregulates the TRPM1 gene expression and also miR211-5p transcription, resulting in the activation of anti-apoptotic molecules and in the survival of ME cells [70].

Further research is required to confirm a direct regulatory effect of miRNA on their potential TRP target genes and to the development of miRNA-based therapy.

Data relative to the effects of TRPM channel-related effects are synthetized in Table 2.

## 4. TRPV Channels in Cancer Therapy

TRPV1 is a Ca^2+^-permeable channel gated by oxidative stress and capsaicin (CPS) and inhibited by the TRPV1 blocker capsazepine (CPZ).

Targeting BC cells with MRS1477, a dihydropyridine derivative acting as a positive allosteric modulator of TRPV1 channels, induces apoptotic cell death. MRS1477 (2 μM) evokes Ca^2+^signals in MCF-7 BC cells, but not in primary breast epithelial cells. Incubation with CPS (10 μM) for 72 h increases ROS production, caspase activity, and apoptosis of BC cells. These effects are further increased when cells are incubated with MRS1477 alone or in combination with CPS. The effects are TRPV1 specific, since CPZ inhibits both the effect of CPS and MRS1477. However, the tumor growth in MCF-7 tumor-bearing immunodeficient mice is not inhibited by MRS1477, suggesting that in vivo further studies are required [72].

In the same view, treatment of MCF-7 BC cells with cisplatin and/or alpha-lipoic acid (ALA) by activating TRPV1 increases [Ca^2+^]i levels, ROS production, lipid peroxidation, mitochondrial membrane depolarization, PARP-1, caspase activation, and apoptosis, and these effects are decreased by CPZ [73].

Recently, static magnetic field application together with CPS (50 μM) has been found to increase its anticancer effects in HepG2 cancer cells by enhancing the CPS-induced mitochondrial-dependent apoptosis. These synergistic effects could be the result of an increased binding efficiency of CPS to TRPV1, induced by a static magnetic field [74].

In the human PC-3 PCa cell line, CPS (20 μM) induces a [Ca^2+^]i increase that is antagonized by CPZ [75]. Moreover, CPS inhibits the DNA synthesis and increases the apoptotic bodies number. Addition of CPZ does not reduce CPS-induced apoptosis but stimulates apoptosis in a similar manner. Both CPS and CPZ increase the production of ROS and mitochondrial potential (ΔΨm) dissipation, suggesting that oxidant stress induced by vanilloids in PC-3 cells is a TRPV1-independent effect. CPS and CPZ further induce caspase-3 activation and reduce tumor growth in vivo. Thus, vanilloids could be used as pharmacological tools against hormone-refractory PCa; however, the contribution of TRPV1 as a potential target is still unclear [75].

The TRPV1 channel has been reported to be the main target of CPS-induced apoptosis in GBM. In the GBM cell line U373, CPS (50 μM) increases [Ca^2+^]_i_ and induces p38 activation, ΔΨm dissipation, and caspase-3 activation, leading to apoptosis. All these effects are reverted by CPZ. Interestingly, the TRPV1 expression is inversely correlated with the grade in GBM, suggesting that TRPV1 may negatively control cancer progression. The loss of TRPV1 in high-grade GBM may represent a mechanism by which cancer cells can evade anti-proliferative and pro-apoptotic signals [76].

Stock et al. showed that in high-grade astrocytomas (AS), neural precursor cells, by releasing endovanilloids that activate TRPV1 channels in cancer cells, induce cell death [77]. Treatment of high-grade AS with arvanil (50 nM) induces a TRPV1-dependent cell death as a consequence of endoplasmic reticulum (ER) stress triggering and TRPV1 expression in the ER membrane. CPZ reversed cell death in high-grade AS in a TRPV1-dependent manner.

In BCa, the TRPV1 agonist CPS (80 μM) promotes Fas/CD95-mediated apoptosis [78]. CPS reduces in a dose-dependent manner the proliferation of the human well-differentiated low-grade papillary RT4 BCa cell line. Moreover, CPS induces the upregulation of pro-apoptotic genes as well as TRPV1-Fas/CD95 receptor clustering and activation, which trigger both extrinsic and intrinsic mitochondrial-dependent pathways. Importantly, all of these effects are reversed by CPZ. Similarly, to GBM, TRPV1 expression decreased in invasive BCas with a complete loss of TRPV1 in high-grade cancers [78]. Moreover, a more aggressive gene phenotype and invasiveness has been evidenced in BCa cells lacking TRPV1 [79].

The agonist CPS evokes Ca^2+^influx in etoposide-resistant but not in etoposide-sensitive WERI-Rb1 retinoblastoma cells [80]. A recent report [81] shows the synergistic effect of CPS in combination with the tyrosine kinase inhibitor, sorafenib, in HCC patients. The drug combination exerts a potent anti-tumor effect by suppressing the EGFR and the PI3K/Akt/mTOR signaling pathways. At present, the contribution of TRPV1 to the CPS-mediated effect has not been addressed so far.

TRPV1 is also involved in increasing the chemosensitivity of cisplatin induced by ALA in BC cells. ALA administration, through TRPV1 activation, increases cisplatin-induced apoptosis, stimulating mitochondrial membrane depolarization, ROS production, lipid peroxidation, caspase-3 and -9 expression, and the first responder of DNA damage, PARP-1. ALA-dependent stimulation of TRPV1 enhances oxidative stress, making BC cells more sensitive to the action of chemotherapeutic drug [73]. TRPV1 channels are sensitive to endocannabinoid exposure, which suppresses cell invasion. Indeed, Ramer et al. showed that low concentrations of R(+)- methanandamide decreased cell invasion and this effect was reversed by CPZ in human cervical cancer cells (HeLa, C33A) as well as human lung carcinoma cells (A549) [82].

Finally, CPZ has been studied as a potential pharmacological tool in cancer. Indeed, at the 50 nM concentration, CPZ sensitizes CRC cells to apoptosis stimulated by tumor necrosis factor (TNF)-related apoptosis-induced ligand [83].

The role of TRPV2 in cancer is still unclear. While it has been described as a regulator of stem-like cell differentiation and chemotherapeutics uptake in GBM, it is also associated with the metastatic status of PCa and BCa, where it stimulates cell migration and invasion.

Cannabidiol (CBD) is a non-psychoactive cannabinoid with anti-tumor activities, acting as a TRPV2 agonist. Nabissi et al. showed that CBD (about 20 μM) increased drug uptake and potentiated cytotoxic activity in GBM when co-administered with cytotoxic agents [84]. Moreover, TRPV2 has been shown to promote, both in vitro and in vivo, differentiation of glioma stem-like cells, leading to a decreased proliferation rate [85].

Over-expression of TRPV2 in GBM increases the sensitivity to Fas/CD95 and Carmustine (BCNU)-induced cytotoxicity [84,85]. In addition, CBD-induced TRPV2 activation reduced BCNU resistance in GBM cells. In fact, CBD inhibits the Ras/Raf/MEK/ERK pathway and promotes drug retention in GBM cells, by restoring the chemoresistant phenotype, improving the apoptosis induced by temozolomide (TMZ), BCNU, and doxorubicin. Mutations of the TRPV2 pore completely inhibit CBD-induced chemoresistance. CBD, through TRPV2 activation, stimulates autophagy in glioma stem-like cells, promoting cell differentiation, and increasing the sensitivity to BCNU- and TMZ-mediated apoptosis [85].

Furthermore, in MM cells, CBD induces TRPV2 upregulation and enhances the sensitivity to Bortezomib, improving cell growth inhibition, cell cycle arrest at the G1 phase, and mitochondrial and ROS-dependent necrosis, mainly in TRPV2-transfected RPMI8226 and U266 MM cells.

TRPV2 activation by CBD (20 μM) decreases proliferation and increases susceptibility to the proteasome inhibitor, bortezomib (3 ng/mL)-induced cell death in human MM cells. Previous studies have found the presence of heterogeneous CD138^+^TRPV2^+^ and CD138^+^TRPV2^-^ plasma cell subpopulations in MM patients. CBD, itself or in synergy with bortezomib, is able to inhibit growth, arrest cell cycle progression, and induce MM cell death by regulating the ERK, Akt, and NF-κB pathways with major effects in CD138^+^TRPV2^+^ MM cells [86,87].

It has also been shown that endogenous lysophospholipids, including lysophosphatidylcholine and lysophosphatidylinositol, enhance the migration of human PCa (PC-3) cells via Ca^2+^ influx through TRPV2 channels by promoting its translocation to the membrane [88]. Similarly, adrenomedullin promote migration and invasion of PC-3 and T24/83 UC cells, through TRPV2 translocation to the plasma membrane, increase resting Ca^2+^ levels [89].

Recent reports suggest that TRPV2 is involved in the maintenance of cancer stem cells (CSCs). By microarray analysis, TRPV2 is found to be upregulated in CSCs. Tranilast, a TRPV2 inhibitor, at 50 μM, suppress OSCC stem cells, generated from ALDH1A1-positive OSCC TE8 cells, more than non-CSC and decreases tumor sphere numbers. Furthermore, tranilast reduces the cell population, which express ALDH1A1 among TE8 cells, suggesting a potential role as a targeted therapeutic agent against cisplatin-resistant OSCC, since CSCs, generated from TE8 cells, are resistant to cisplatin and have the ability to re-differentiate [90]. In addition to TRPV2, tranilast is also a weak TRPM2 inhibitor [91].

The antimicrobial peptide hCAP18/LL-37 stimulates cell migration and metastasis in several cancers. In BC, LL-37 (2 mM) induces migration by activating the TRPV2 and recruiting it to pseudopodia in a PI3K/AKT-dependent manner. Ca^2+^entry, through TRPV2, cooperates with a K^+^ efflux through the large conductance Ca^2+^-activated K^+^ (BKCa) channels. However, LL-37 attaches to the membranes of caveolae and pseudopods and decreases the fluidity of the membrane, suggesting it as a mechanism of changes in the physical properties of the bilayer lipid membrane [92], instead of TRPV2-specific binding.

The TRPV6 channel is overexpressed in different cancers [93]. Soricidin and its derivatives (SOR-C13, 14 nM; and SOR-C27, 65 nM) inhibit TRPV6-dependent Ca^2+^ uptake and bind TRPV6 with a high affinity in ovarian cancer (OC) [94]. Soricidin is a 54-amino acid peptide found in the paralytic venom of the short-tailed shrew *Blarinabrevicauda.* Recently, Bowen et al. described the use of the TRPV6-binding properties of SOR-C13 and SOR-C27 to target human OCs in a xenograft mouse model [94]. In addition, the side effects and best dose as well as safety/tolerability, pharmacokinetics, pharmacodynamics, and efficacy in treating patients with solid tumors, were evaluated in two different clinical trials (NCT03784677, NCT01578564, respectively). The first trial is in the recruiting stage (clinicaltrials.gov accessed 4 November 2019), whereas the data of the second one trial has been recently published. These data show that the administration of SO-C13, up to 6.2 mg/kg, induces in 54.5% of patients (*n* = 22 patients) a stable disease, ranging from 2.8 to 12.5 months, and the best response was a 27% reduction in PC, with a 55% reduction in CA19-9 marker levels [95]. TRPV6 has been shown to mediate the CPS-induced apoptosis (at 50 μM dose) in GaC cells [96] and, interestingly, GaC cells are more sensitive to CPS-induced apoptosis than normal gastric cells. The pretreatment with CPZ prevents CPS-induced TRPV6 expression and apoptosis of GaC cells. The mechanism involved in CPS-induced apoptosis of AGS cells depends on increased Ca^2+^ influx via TRPV6 channels; moreover, CPS induces apoptosis by stabilization of p53 through c-Jun N-terminal kinases activation.

Calcitriol, in combination with dietary soy, enhances anticancer activity and increases hypercalcemic toxicity in a mouse xenograft model of PCa. This combination upregulates the expression of anti-proliferative (p21, IGFBP-3) and pro-apoptotic (Bax) genes, increasing the inhibition of anti-apoptotic (Bcl-2) and cell cycle-promoting (cyclin D1) genes, thus suppressing prostaglandin (PG) synthesis and signaling (COX-2, 15-PGDH). These effects seem to be TRPV6-dependent; in fact, the calcium increase in serum is associated with elevated TRPV6 and calbindin-9k, an intestinal calcium absorption gene [97].

The TRPV6 channel is upregulated in BC cell lines and in BC samples compared to normal tissue. TRPV6 overexpression is associated with reduced OS in estrogen receptor-negative BCs as well as HER2-positive tumors. Downregulation of TRPV6 expression reduces basal Ca^2+^influx, leading to a reduction in cell proliferation and DNA synthesis [98]. In *Xenopus* oocytes transfected with TRPV6, tamoxifen inhibits Ca^2+^uptake and the expression of TRPV6 at mRNA levels in BC cell lines. The silencing of TRPV6 improves the pro-apoptotic activity of tamoxifen, suggesting that the increase of Ca^2+^influx, mediated by TRPV6 overexpression in BCs, is responsible for the reduced sensitivity to tamoxifen treatment [99]. Tamoxifen is also able to inhibit TRPV6 activity through estrogen receptor-independent pathways in TRPV6-overexpressing MCF-7 BC cells. Tamoxifen reduces the basal intracellular Ca^2+^concentration and this inhibitory effect is not blocked by the estrogen receptor antagonist, ICI 182,720. The effect of tamoxifen is completely blocked by activation of protein kinase C (PKC). After inhibiting the PKC with calphostin C, the activity of TRPV6 is decreased but does not alter the effect of tamoxifen, suggesting that the therapeutic effect of tamoxifen and PKC inhibitors might lead to the entry of TRPV6-mediated calcium [100].

The expression level of TRPV6, assessed using laser capture microdissection, is higher in invasive BC tissue than in the non-invasive one. In addition to MCF-7 cell migration inhibition, TRPV6 silencing can inhibit MDA-MB-231 migration and invasion [27]. Moreover, limited estrogen receptor signaling leads to lower levels of TRPV6 expression and the antitumor effect of tamoxifen seem to be related to inhibition of TRPV6 channel expression [101].

Data relative to the effects of TRPV channel-related effects are synthetized in Table 3.

## 5. TRPA1 Channels in Cancer Therapy

Recent evidence shows that TRPA1 is involved in chemotherapy-induced pain syndrome. Thus, dacarbazine (DBZ), used mainly to treat metastatic melanoma, is reported to cause painful symptoms. Experiments using mouse dorsal root ganglion neurons and human TRPA1-transfected HEK293 (hTRPA1-HEK293) cells as well as naïve mice and TRPA1-knockout mice (1 mg/Kg/day) and B16-F10 melanoma cells treated with DBZ, demonstrated that DBZ directly activates TRPA1 and sensitizes it indirectly by generating oxidative stress products. Moreover, DBZ causes mechanical and cold allodynia in naïve but not in TRPA1-knockout mice. Pharmacological blockade of TRPA1 also reduces DBZ-induced nociception in a tumor-associated pain model [102].

On the other hand, in GBM cells, it has been shown that inhibition of TRPA1 is not a good strategy to kill cancer cells. Indeed, the ALA at 50 μM inhibits the TRPA1 channel in human GBM (DBTRG) cells, attenuating hypoxia-induced apoptosis, inflammation, and TRPA1-mediated mitochondrial oxidative stress [103].

Resveratrol induces activation of mutated TRPA1 in human PCa-associated fibroblasts. Previous studies have shown the effects of Resveratrol (6.5 μM) in inducing apoptosis in PCa cells without taking into consideration its impact on the tumor microenvironment. Recently, it has been reported that resveratrol activates N-terminal-mutated TRPA1, leading to intracellular calcium increase, and increases HGF and VEGF expression and secretion, without inducing apoptosis in these cells [104].

The steroidal and non-steroidal third-generation aromatase inhibitors, exemestane, letrozole and anastrozole, used in BCs, induce pain-like symptoms through TRPA1, suggesting that TRPA1 antagonists can be used for the treatment of pain associated with aromatase inhibitors [105]. Methyl syringate (100 μM), a TRPA1 agonist, has been reported to repress hypoxia-induced cyclooxygenase-2 (COX-2) in lung A549 cancer cells [106]. Methyl syringate suppresses in a TRPA1-dependent manner hypoxia-induced COX-2 and promoter activity and reduced hypoxia-induced migration and invasion and secretion of vascular endothelial growth factor.

Stimulation of TRPA1 by allyl isothiocyanate (AITC), at 10 mM, promotes cell survival of small cell lung cancer cells (SCLCs) [107]. TRPA1 mRNA is significantly expressed in SCLC patients as compared to non-SCLC samples or non-malignant lung tissue. Stimulation of SCLC cells with AITC leads to a rise of [Ca^2+^]_i_. Furthermore, AITC stimulates ERK in TRPA1-expressing HEK293 cells and in SCLC cells via an Src- and calcium-dependent mechanism. In addition, TRPA1 activation in SCLC cells prevents apoptosis and induces survival in a TRPA1- and ERK-dependent manner. On the contrary, downregulation of TRPA1 impairs the growth of SCLC cells. These data suggest that exogenous inhalable activators of TRPA1 exert tumor-promoting effects in SCLC cells. Finally, blockade of TRPA1 by its antagonist HC-030031 prevents chemotherapy-induced peripheral neuropathy [108].

Data relative to the effects of TRPA1 channel-related effects are synthetized in Table 4.

## 6. Conclusions

Despite advances in the detection of more specific therapies for various tumors, high rates of treatment failure and mortality still exist. These high rates depend on the ability of tumors to progress from local to systemic disease. TRP channels are Ca^2+^-selective ion channels involved in cancer development and metastatic spreading by controlling different stages of tumor progression. Several pharmacological approaches targeting the TRP channels in cancer have been employed. However, the major obstacle in evaluating the effects of TRP agonists/antagonists and inhibitors/activators in cell lines or in other experimental models is due with their reduced specificity. Although most of these studies have provided interesting data on the cellular and molecular mechanisms driving tumor progression, the development of new specific drugs targeting TRP channels is only just beginning. In addition, although it is well known that the expression of TRP channels is altered during cancer progression, the TRP signaling pathways conditioning the behaviors of cancer cells as well as the pharmacological response are still poorly elucidated.

In this regard, previously, we demonstrated that ERK-induced TRPV2 activation by treatment with TRPV2 agonist, CBD, overcomes drug resistance in GBM [84,109]. Moreover, recently, it has been reported that the endolysosomal TRPML1 channel, belonging to the mucolipin TRP channel family, is required for cancer cell proliferation bearing HRAS mutations and that TRPML1 expression was significantly elevated in HRAS-positive tumors and inversely correlated with poor prognosis. Mutations of KRAS and HRAS in cancer affect the efficacy of chemotherapy; changes in TRP channel expression could overcome drug resistance and increase the sensitivity of cancer cells to chemotherapy [110]. The mucolipin TRPML1 receptor is also required in ME cells to negatively regulate MAPK and mTORC1 signaling [111] and in TNBC, where it regulates cancer development by promoting mTORC1 and purinergic signaling pathways [112].

Overall, we are only beginning to develop drugs specifically targeting TRP channels and TRP-mediated signaling pathways.

Furthermore, the role of changes in the epigenetics of TRP channels in cancers should also be considered. It has been demonstrated that inactivation of *TRP* genes by aberrant methylation (hyper- and hypo-methylation) of GC-rich DNA regions, CpG islands, is suggested to be involved in tumor development and progression. In this regard, hydrogen peroxide has been found to induce demethylation of the TRPM2 promoter region and increase the expression of TRPM2 in melanocytes [113]; the TRPM6 and TRPM7 ion channels have been found to bind to the chromatin-remodeling complexes, induce histone phosphorylation, and decrease arginine methylation, resulting in changes in the transcription of hundreds of genes [114,115]; transactivation of TRPA1 promoter by Notch1 receptor intracellular domain, which promotes TRPA1 expression in erytroleukemic cells, suppresses erythroid differentiation [116]. Moreover, regarding cancer chemoresistance, miR-320a is a mediator of the chemoresistance of BC cells by targeting TRPC5 and NFATc3 and the expression of miR320a is regulated by methylation of its promoter and the transcription factor, v-ets erythroblastosis virus E26 oncogene homolog 1 [117].

Aberrations in the expression of different splice variants, and their expression during tumor progression, may trigger a variety of Ca^2+^ signaling, which may contribute to the generation of more aggressive tumor clones. Thus, in PCa cells, two short splice variants of TRPM8, named TRPM8α and TRPM8β, with reducing activity, have been identified [118], and two different TRPM1 isoforms have been reported in human ME [119]. Finally, in BCa, an alternative splice variant of TRPV2 that acts as a dominant negative mutant of wild-type TRPV2 has been identified [120].

Epigenetic changes of TRP channels could be the rationale to approach a personalized therapy in cancer patients. Further studies are required to completely achieve this important issue. Deeper studies on TRP ion channels in cancer are required in order to have a major impact on the development of new drugs targeting the TRP signaling network and TRP isoforms in cancer cells.

## Figures and Tables

**Table 1:** Pharmacological modulation of TRPC channel expression and functions by natural and/or chemical agents in cancer cells.

Pharmacological Agent	TRP Target	Cancer Type	Effects	Mechanisms	References
20-GPPD	TRPC (?)	CRC	Cell apoptosis (+)	Ca^2+^ entry ↑	[11]
SKF96365/2-APB/MRS1845	TRPC1	GBM	Cell growth (−)	SOCE ↓	[13]
SKF96365/2-APB/MRS1845	TRPC1	GBM	Cell migration (−)	SOCE ↓	[14]
MTI-101	TRPC1	MM	Necrosis (+)	Ca^2+^ entry ↑	[22]
SKF96365/2-APB	TRPC1, C3, C4, C5	GBM	Cell growth (−)	Cytokinesis ↑	[12]
ATRA	TRPC3, C4, C6	NSCLC	Proliferation (−)	Ca^2+^ entry ↑	[23]
EA	TRPC4/C5	RCC	Cell proliferation (−)	Ca^2+^ entry ↑	[16]
	TRPC1/4/5	SSC	Cytotoxicity (+)	Na^+^ entry ↑	[19]
TZL	TRPC1/4/5	RCC	Cytotoxicity (+)	PKC θ activity ↑	[20]
Nicotine	TRPC1/C6	NSCLC	Proliferation (+)	HIF-1α and SOCCs ↑	[24]
AM237	TRPC5	RCC	Viability (+)	Sphingosine-1-phosphate activity ↑	[21]
SKF96365	TRPC6	GCa, OSCC	Cell growth (−)	Ca^2+^ entry ↑	[31,32]

Abbreviations: 2-APB, 2-Aminoethoxydiphenyl borate; 20-GPPD, 20-*O*-β-d-glucopyranosyl-20(S)-protopanaxadiol; ATRA, all-trans retinoic acid; GBM, glioblastoma; BC, breast carcinoma; CRC, colorectal carcinoma; HIF-1α, hypoxia Inducible Factor 1α; EA, Englerin A; GCa, gastric carcinoma; MM, multiple myeloma; NSCLC non small lung cell carcinoma; OSCC, eosophageal carcinoma; RCC, renal cell carcinoma; SOCE, Store Operated Calcium Entry; SOCCs, Store Operated Calcium Channels; SSC, synovial sarcoma cell. (?) no conclusive data. (↑) increment. (↓) reduction. (+) increase. (−) decrease.

**Table 2:** Pharmacological modulation of TRPM channel expression and functions by natural and/or chemical agents in cancer cells.

Pharmacological Agent	TRP Target	Cancer Type	Effects	Mechanisms	References
Tricostatin A/ Sodium butirrate	TRPM2	BCa	Apoptosis (+)	Ca^2+^ entry↑	[71]
5FU/Leucoverin	TRPM2	BC, CRC	Apoptosis (+)	Ca^2+^ entry ↑	[35]
Se/Docetaxel	TRPM2	GBM	Apoptosis (+)	ROS generation ↑ Ca^2+^ entry ↑	[36]
WA	TRPM7	LK	Cell proliferation (-)	Mg^2+^-dependent TRPM7 block ↑	[39]
WA	TRPM7	BC, GCa	Cell proliferation (-)	Mg^2+^-dependent TRPM7 block ↑	[40]
Ginseroside Rd	TRPM7	GCa, BC	Apoptosis (+)	TRPM7 currents ↑	[41]
TG100-115 activity ↓	TRPM7	BC	Cell migration (-) Invasion (-)	TRPM7 Kinase	[38]
WS-12	TRPM8	PCa	Migration (-)	ND	[51,52]
Menthol	TRPM8	BCa	Cell viability (-) and death (+)	Mitochondrial depolarization ↑	[53]
BCTC	TRPM8	PCa	Cell proliferation (-)	Ca^2+^ entry ↓	[56]
Clotrimazole	TRPM8	PCa	Cell proliferation (-)	Ca^2+^ entry ↓	[56]
DD01050	TRPM8	PCa	Cell proliferation (-)	Ca^2+^ entry ↓	[56]
AMTB TRPM8		PCa	Cell proliferation (-)	ND	[57]
JNJ41876666 TRPM8		PCa	Cell proliferation (-)	ND	[57]
RQ	TRPM8	OSC	Cell migration and Invasion (-)	SOCE and MMP-9 activity ↓	[54]
CBG	TRPM8	CRC	Cell growth (-) Apoptosis (-)	Endocannabinoids reuptake ↑	[55]
Thisoquinoline-derived Urea 2,5’DKpiperazine derivates	TRPM8	PCa	Viability, cell growth and apoptosis (-)	Ca^2+^ entry ↓	[58]
Triphanyphosphine oxide TRPM5		ME	Lung metastasis (-)	ETA-induced [Ca^2+^]i ↓ MMP9 ↑	[37]

Abbreviation: 5-FU, fluorouracil; BC, breast carcinoma; BCa, bladder cancer; CBG, cannabigerol; CRC, colorectal carcinoma; ETA, extracellular acid; GBM, glioblastoma; GCa, gastric carcinoma; ME, melanoma; MMP-9, Metallo-proteases 9; OSC, oral squamous carcinoma; PCa, prostate carcinoma; ROS, reactive oxygen species; RQ, RQ-0023078; Se, Selenium; SOCE, Store Operated Calcium Entry; TRPM7, transient receptor potential melastanin 7; WA, Waixenicin A. ND, not detected. (↑) increment. (↓) reduction. (+) increase. (−) decrease.

**Table 3:** Pharmacological modulation of TRPV channel expression and functions by natural and/or chemical agents in cancer cells.

Pharmacological Agent	TRP Target	Cancer Type	Effects	Mechanisms	References
CPS	TRPV1	PCa	Cell growth (−) and apoptosis (+)	[Ca^2+^] ↑ Oxidative stress ↑	[75]
CPS	TRPV1	GBM	Apoptosis (+)	Ca^2+^ entry ↑	[76]
CPS	TRPV1	BCa	Cell growth (−)	[Ca^2+^]i↑	[78]
Arvanil	TRPV1	AS	Apoptosis (+)	ER stress ↑	[77]
ALA/cisplatin	TRPV1	BC	ROS-induced apoptosis (+)	[Ca^2+^] ↑	[73]
CPS /MRS1477	TRPV1	BC	Cell viability (−) and apoptosis (+)	Oxidative Stress ↑	[72]
CPS /SM field	TRPV1	HCC	Apoptosis (+)	Binding affinity ↑	[74]
CPS /Sorafenib	TRPV1 (?)	HCC	Invasion (−) and autophagy (+)	AMPK activity ↓	[81]
CBD	TRPV2	GBM	Apoptosis (+) and drug sensitization (+)	Ca2+ entry ↑	[84]
CBD/Bortezomib	TRPV2	MM	Cell proliferation (−) and Drug sensitivity (+)	ERK activity ↓	[86]
LL-37 peptides	TRPV2 and BKCa	BC	Cell migration (+)	Ca^2+^ and K^+^ entry ↑	[92]
Tranilast	TRPV2	OSCC	Cytotoxicity (+)	Stemness ↓	[90]
CPS	TRPV6	GCa	Apoptosis (+)	Ca^2+^ entry ↑	[96]
Tamoxifen	TRPV6	BC	Transport Ca^2+^ rate (−)	PKC inhibition ↑	[100]
Calcitriol	TRPV6	BC	Cell growth (+)	Ca^2+^ entry ↑	[93]
	TRPV6	LK	Cell differentiation (+)	Ca^2+^ entry (?)	[93]
SOR-C13/SOR-C27	TRPV6	OC, PCa	Cell proliferation (−)	[Ca^2+^]i ↓	[94]

Abbreviation: ALA, Lipoich acid; AS, astrocytoma; GBM, glioblastoma; BC, breast carcinoma; BKCa, large-conductance voltage-and Ca^2+^ activated K+ channel; CBD, cannabidiol; CPS, Capsaicin; ER, endoplasmatic reticulum; ERK, extracellular signal–regulated kinases; GCa, gastric carcinoma; HCC, hepatocarcinoma; MM, multiple myeloma; OC, ovarian carcinoma; OSCC, eosophageal squamous carcinoma; PCa, Prostate carcinoma; Se, selenium; SM field, static magnetic field. (↑) increment. (↓) reduction. (+) increase. (−) decrease. (?) no conclusive data.

**Table 4:** Pharmacological modulation of TRPA channel expression and functions by natural and/or chemical agents in cancer cells.

	TRP Target	Cancer Type	Effects	Mechanisms	References
Dacarbazine	TRPA1	ME	Hypersensitivity (+)	Oxidative stress ↑	[102]
ALA	TRPA1	GBM	Apoptosis (−)	Mitochondrial oxidative stress ↓	[103]
Res	TRPA1	PCa	Apoptosis (−)	Growth Factor release ↑	[104]
Exemestane, Letrozole Anastrozole (Als)	TRPA1	BC	Nociception and allodynia (+)	Ca^2+^ entry ↑	[105]
Methyl syringate	TRPA1	NSCLC	Migration and invasion (−) VEGF (−)	Hypoxia induced COX-2/PGE2 activity ↓	[106]
AITC	TRPA1	NSCLC	Apoptosis (−)	Ca^2+^ entry ↑	[107]

Abbreviations: ALA, Alpha Lipoic acid; Als, aromatase inhibitors; AITC, Allyl isothiocyanate; BC, breast carcinoma; GBM, glioblastoma; ME, melanoma; NSCLC, non small cell lung carcinoma; OSC, oral squamous carcinoma; PCa, prostate carcinoma; Res, Resveratrol. (↑) increment. (↓) reduction. (+) increase. (−) decrease.

## Abbreviations

2-APB	2-Aminoethoxydiphenyl borate
20-GPPD	20-*O*-β-d-glucopyranosyl-20(S)-protopanaxadiol
5-Fu	5-Fluorouracil
ADMR	adriamycin resistant
AITC	allyl isothiocyanate
AKT	protein kinase B
ALA	α-lipoic acid
AMPK	AMP-activated protein kinase
AR	androgen receptor
AS	astrocytoma
ATRA	all-trans retinoic acid
BC	breast cancer
BKCa	large-conductance voltage-and Ca^2+^activated K^+^channel
CBD	cannabidiol
CBG	cannabigerol
ccRCC	clear cell renal cell carcinoma
CHOP	CCAAT-enhancer-binding protein homologous protein
CPS	capsaicin
CPZ	capsazepine
CRC	colorectal cancer
CSC	cancer stem cell
DBZ	dacarbazine
DC	dendritic cell
DTX	docetaxel
EA	Englerin A
EGF	epidermal growth factor
EMT	epithelial mesenchymal transition
ERK	extracellular signal–regulated kinases
EV	extracellular vessicle
GaC	gastric cancer
GBM	glioblastoma
GSK-3β	glycogen synthase kinase-3β
HCC	hepatocellular carcinoma
HIF-1α	hypoxia Inducible Factor 1α
IP_3_LCV	inositol triphosphateleucovorin
ME	melanoma
MEL	melatonin
miRNA	microribonucleic acid
MM	multiple myeloma
MMP-9	matrix metalloproteinase-9
MITF	microphthalmia-associated transcription factor
NFATc3	nuclear factor of activated T-cells 3
NSCLC	non-small cell lung cancer
OC	ovarian carcinoma
OS	overall survival
OSCC	eosophageal carcinoma
OSC	oral squamous carcinoma
PARP-1	poly (ADP-ribose) polymerase-1
PC	pancreatic cancer
PCa	prostate cancer
PG	prostaglandin
P-gp	P-glycoprotein
PI3K	phosphatidylinositole–3-kinase
PKC	protein kinase C
PSA	prostate-specific antigen
RACK1	receptor of activated protein C kinase 1
RCC	renal cell carcinoma
RES	resveratrol
ROS	reactive oxigen species
Se	selenium
SSC	synovial sarcoma cell
SOCC	store operated calcium channels
SOCE	store-operated calcium entry
TNBC	triple-negative breast cancer
TRP	transient potential receptor
TRPA	transient potential receptor ankyrin
TRPC	transient potential receptor canonical
TRPM	transient potential receptor melastatin
TRPML	transient potential receptor mucolipidin
TRPP	transient potential receptor polycystic
TRPV	transient potential receptor vanilloid
TZL	tonantzitlolone
Vac	vacquinol-1
VHL	von Hippel-Lindau
WA	waixenicin A
